# Severe Acute Respiratory Syndrome Coronavirus Viroporin 3a Activates the NLRP3 Inflammasome

**DOI:** 10.3389/fmicb.2019.00050

**Published:** 2019-01-29

**Authors:** I-Yin Chen, Miyu Moriyama, Ming-Fu Chang, Takeshi Ichinohe

**Affiliations:** ^1^Division of Viral Infection, Department of Infectious Disease Control, International Research Center for Infectious Diseases, Institute of Medical Science, The University of Tokyo, Tokyo, Japan; ^2^Institute of Biochemistry and Molecular Biology, National Taiwan University College of Medicine, Taipei, Taiwan

**Keywords:** SARS-CoV, viroporin, inflammasome, IL-1β, inflammation

## Abstract

Nod-like receptor family, pyrin domain-containing 3 (NLRP3) regulates the secretion of proinflammatory cytokines interleukin 1 beta (IL-1β) and IL-18. We previously showed that influenza virus M2 or encephalomyocarditis virus (EMCV) 2B proteins stimulate IL-1β secretion following activation of the NLRP3 inflammasome. However, the mechanism by which severe acute respiratory syndrome coronavirus (SARS-CoV) activates the NLRP3 inflammasome remains unknown. Here, we provide direct evidence that SARS-CoV 3a protein activates the NLRP3 inflammasome in lipopolysaccharide-primed macrophages. SARS-CoV 3a was sufficient to cause the NLRP3 inflammasome activation. The ion channel activity of the 3a protein was essential for 3a-mediated IL-1β secretion. While cells uninfected or infected with a lentivirus expressing a 3a protein defective in ion channel activity expressed NLRP3 uniformly throughout the cytoplasm, NLRP3 was redistributed to the perinuclear space in cells infected with a lentivirus expressing the 3a protein. K^+^ efflux and mitochondrial reactive oxygen species were important for SARS-CoV 3a-induced NLRP3 inflammasome activation. These results highlight the importance of viroporins, transmembrane pore-forming viral proteins, in virus-induced NLRP3 inflammasome activation.

## Introduction

Severe acute respiratory syndrome coronavirus (SARS-CoV), a member of the genus *Betacoronavirus* within the family *Coronaviridae*, is an enveloped virus with a single-stranded positive-sense RNA genome of approximately 30 kb in length. The 5′ two-thirds of the genome encodes large polyprotein precursors, open reading frame (ORF) 1 and ORF1b, which are proteolytically cleaved to generate 16 non-structural proteins (Tan et al., 2005). The 3′ one-third of the genome encodes four structural proteins, spike (S), envelope (E), matrix (M) and nucleocapsid (N), and non-structural proteins, along with a set of accessory proteins (3a, 3b, 6, 7a, 7b, 8a, 8b, and 9b) (Perlman and Dandekar, 2005; Tan et al., 2005). SARS-CoV is the etiological agent of SARS (Drosten et al., 2003; Fouchier et al., 2003; Ksiazek et al., 2003; Kuiken et al., 2003; Peiris et al., 2003). At least 8,098 laboratory-confirmed cases of human infection, with a fatality rate of 9.6%, were reported to the World Health Organization from November 2002 to July 2003. High levelsof proinflammatory cytokines, including tumor necrosis factor (TNF)-α, interleukin (IL)-1β, and IL-6, were detected in autopsy tissues from SARS patients (He et al., 2006). Although dysregulation of inflammatory cytokines may be involved in lung injury and the pathogenesis of SARS-CoV, the underlying molecular mechanisms are not fully understood.

The innate immune systems utilizes pattern recognition receptors (PRRs) to detect pathogen-associated molecular patterns (Medzhitov, 2001; Kawai and Akira, 2010). Recognition of virus infection plays an important role in limiting virus replication at the early stages of infection. Nod-like receptor family, pyrin domain-containing 3 (NLRP3) is activated by a wide variety of stimuli, including virus infection (Bauernfeind et al., 2011). Four models describing activation of the NLRP3 inflammasome have been proposed thus far (Hornung and Latz, 2010; Schroder et al., 2010; Tschopp and Schroder, 2010). First, the disturbances in intracellular ionic concentrations, including K^+^ efflux and Ca^2+^ influx, play an important role (Fernandes-Alnemri et al., 2007; Petrilli et al., 2007; Arlehamn et al., 2010; Ichinohe et al., 2010; Ito et al., 2012; Murakami et al., 2012; Munoz-Planillo et al., 2013). Second, cathepsin B and L, which are specific lysosomal cysteine proteases, are though to play a role after phagocytosis of cholesterol crystals (Duewell et al., 2010), fibrillar peptide amyloid-beta (Halle et al., 2008), silica crystals, and aluminum salts (Hornung et al., 2008). Third is the release of reactive oxygen species (ROS) or mitochondrial DNA from damaged mitochondria (Zhou et al., 2010, 2011; Nakahira et al., 2011; Shimada et al., 2012). Finally, viral RNA or RNA cleavage products generated by RNase L activate the NLRP3 inflammasome via the DExD/H-box helicase, DHX33 (Allen et al., 2009; Mitoma et al., 2013; Chen et al., 2014; Chakrabarti et al., 2015). Upon activation, the NLRP3 is recruited to the mitochondria via association with mitochondrial antiviral signaling (MAVS) or mitofusin 2 expressed on the outer mitochondrial membrane (Ichinohe et al., 2013; Subramanian et al., 2013); these molecules then recruit the apoptosis-associated speck-like protein containing a caspase recruitment domain (ASC) and pro-caspase-1 to form the NLRP3 inflammasome. This event activates the downstream molecule, caspase-1, which catalyzes the proteolytic processing of pro-IL-1β and pro-IL-18 into their active forms and stimulates their secretion (Kayagaki et al., 2015; Shi et al., 2015).

It is increasingly evident that NLRP3 detects RNA viruses by sensing the cellular damage or distress induced by viroporins (Ichinohe et al., 2010; Ito et al., 2012; Triantafilou et al., 2013; Nieto-Torres et al., 2015), transmembrane pore-forming proteins, encoded by certain RNA viruses; these proteins alter membrane permeability to ions by forming membrane channels (Tan et al., 2005; Chen and Ichinohe, 2015). A recent study shows that the SARS-CoV E protein, which comprise only 76 amino acids, forms Ca^2+^-permeable ion channels and activates the NLRP3 inflammasome (Nieto-Torres et al., 2015). Although the E and 3a proteins of SARS-CoV, which comprise 274 amino acids and contain three transmembrane domains (Zeng et al., 2004; Lu et al., 2006), are thought to act as Na^+^/K^+^ and K^+^ channels, respectively (Wilson et al., 2004; Lu et al., 2006; Torres et al., 2007; Parthasarathy et al., 2008; Pervushin et al., 2009; Wang et al., 2011), the role of the 3a protein in activating the NLRP3 inflammasome remains unknown. Here, we examined the role of the 3a protein in activating the NLRP3 inflammasome.

## Materials and Methods

### Mice

Six-week-old female C57BL/6 mice were purchased from The Jackson Laboratory. All animal experiments were approved by the Animal Committees of the Institute of Medical Science (The University of Tokyo).

### Cells and Viruses

Bone marrow-derived macrophages (BMMs) were prepared as described previously (Ichinohe et al., 2009). In brief, bone marrow was obtained from the tibia and femur by flushing with Dulbecco’s modified Eagle’s medium (DMEM; Nacalai Tesque). Bone marrow cells were cultured for 5 days in DMEM supplemented with 30% L929 cell supernatant containing macrophage colony-stimulating factor, 10% heat-inactivated fetal bovine serum (FBS), and L-glutamine (2 mM) at 37°C/5% CO_2_. HEK293FT cells (a human embryonic kidney cell line) and HeLa cells (a human epithelial carcinoma cell line) were maintained in DMEM supplemented with 10% FBS, penicillin (100 units/ml), and streptomycin (100 μg/ml) (Nacalai Tesque). MDCK cells (Madin-Darby canine kidney cells) and HT-1080 cells (a human fibrosarcoma cell line) were grown in Eagle’s minimal essential medium (E-MEM; Nacalai Tesque) supplemented with 10% FBS, penicillin (100 units/ml), and streptomycin (100 μg/ml) (Nacalai Tesque).

Influenza A virus strain A/PR8 (H1N1) was grown at 35°C for 2 days in the allantoic cavities of 10-day-old fertile chicken eggs (Ichinohe et al., 2009). The viral titer was quantified in a standard plaque assay using MDCK cells (Pang et al., 2013).

### Plasmids

cDNAs encoding the E and M proteins of SARS-CoV Frankfurt 1 strain (Matsuyama et al., 2005) were obtained by reverse transcription and PCR of total RNA extracted from SARS-CoV-infected Vero cells, followed by PCR amplification using specific primers. pcDNA3.1D-3a-V5His was provided by Ming-Fu Chang (National Taiwan University College of Medicine, Taipei, Taiwan). To generate the plasmids pLenti6-E-V5His, pLenti6-3a-V5His, and pLenti-M-V5His, cDNA fragments of E, 3a, and M were amplified from pcDNA3.1D-E-V5His, pcDNA3.1D-3a-V5His, and pcDNA3.1D-M-V5His using specific primer sets and then ligated into pLenti6-TOPO vectors (Invitrogen). To generate plasmids pCA7-flag-E, pCA7-flag-3a, and pCA7-flag-M, pCA7-HA-E, pCA7-HA-3a, and pCA7-HA-M, cDNA fragments of E, 3a, and M were amplified from pcDNA3.1D-E-V5His, pcDNA3.1D-3a-V5His, and pcDNA3.1D-M-V5His using specific primer sets, digested with *EcoR* I and *Not* I, and subcloned into the *EcoR* I-*Not* I sites of the pCA7-flag-ASC plasmid or pCA7-HA-M2 plasmid, respectively (Ito et al., 2012). To construct plasmids expressing the E mutant V25F, the mutated E fragments were amplified by inverse PCR with wild-type E-containing plasmids and specific primer sets. The PCR products were cleaved by *Dpn* I, ligated in a ligase- and T4 kinase-containing reaction and then transformed into DH5α competent cells (TOYOBO). To construct plasmids expressing the 3a mutant 3a-CS, fragments were amplified from wild-type 3a-containing plasmids using 3a-specific primer sets and transformed as described above.

### DNA Transfection and Western Blot Analysis

HEK293FT cells were seeded in 24-well cluster plates and transfected with 1 μg pLenti6-E/3a/M-V5His, pLenti-GFP (green fluorescent protein), or pLenti-M2 using polyethylenimine (PEI) Max. At 24 h post-transfection, the cells were lysed with RIPA buffer (50 mM Tris–HCl, 1% NP-40, 0.05% sodium dodecyl sulfate (SDS), 150 mM NaCl and 1 mM EDTA). And the lysates were subjected to SDS-polyacrylamide gel electrophoresis (PAGE) followed by electroblotting onto polyvinylidene difluoride (PVDF) membranes. The membranes were incubated over night with mouse anti-V5-tag (R960-25, Invitrogen), mouse anti-influenza A virus M2 (14C2, Abcam), mouse anti-GFP (GF200, Nacalai Tesque), or rabbit anti-tubulin (DM1A, Santa Cruz) antibodies, followed by horseradish peroxide-conjugated anti-mouse IgG (Jackson Immuno Research Laboratories) or anti-rabbit IgG (Invitrogen). After washing 3 times with washing buffer (0.05% Tween-20/PBS), the membranes were exposed using Chemi-Lumi One Super (Nacalai Tesque), and the chemiluminescent signals were captured by an ImageQuant LAS-4000 mini apparatus (GE Healthcare).

### Lentiviral Vectors

To generate lentiviruses expressing V5-tagged SARS-CoV E, 3a, and M proteins, the full-length cDNA encoding each viral protein was cloned into the pLenti6.3/V5-TOPO vector (Invitrogen) using the following primers: SARS-CoV E forward, 5′-caccatgtactcattcgtttcgga-3′, and reverse, 5′-gaccagaagatcaggaactc-3′; SARS-CoV 3a forward, 5′-caccatggatttgtttatgagatt-3′, and reverse, 5′-caaaggcacgctagtagtcg-3′; SARS-CoV M forward, 5′-caccatggcagacaacggtactat-3′, and reverse, 5′-ctgtactagcaaagcaatat-3′. Sub-confluent monolayers of HEK293FT cells seeded in a collagen-coated dish (10 cm in diameter) were transfected with 3 μg of pLenti6.3/V5-TOPO vector expressing each viral protein or EGFP together with ViraPower Packaging Mix (Invitrogen) using Lipofectamine 2000 (Invitrogen). The supernatants containing lentiviruses were harvested and filtered through a 0.45 μm filter (Millipore) at 72–96 h post-transfection (Ito et al., 2012). The lentiviral titer was then quantified using HT-1080 cells as described previously (Ichinohe et al., 2013).

### Virus Infection

Bone marrow-derived macrophages were plated at a density of 8 × 10^5^ in 24-well plate and infected with A/PR8 influenza virus or lentivirus at a multiplicity of infection (MOI) of 5 or 0.2 for 1 h, respectively. Then, BMMs were stimulated with 1 μg/ml of LPS and cultured for additional 23 h in complete media. Supernatants were collected at 24 h post-infection and centrifuged to remove cell debris. The amount of IL-1β in the supernatants was measured in an enzyme-linked immunosorbent assay (ELISA) using paired antibodies (eBioscience) (Ichinohe et al., 2010, 2013).

### Confocal Microscopy

To clarify the cellular localization of the wild-type and mutant 3a proteins of SARS-CoV, HeLa cells were cultured on coverslips and transfected with 1 μg of pCA7-flag-3a or pCD7-flag-3a-CS together with 0.5 μg of ER-mCherry or DsRed-Golgi (Ito et al., 2012). At 24 h post-transfection, cells were fixed with 4% paraformaldehyde and permeabilized with 1% Triton X-100/PBS. After washing with PBS and blocking with 4% BSA/PBS, the cells were incubated with a mouse anti-flag antibody (M2, Sigma) followed by incubation with Alexa Fluor 488-conjugated goat anti-mouse IgG (H+L) (Life Technologies).

To observe the cellular distribution of NLRP3 in the E- or 3a-expressing cells, HeLa cells were cultured on coverslips and transfected with 1 μg of pCA7-HA-E, pCA7-HA-EV25F, pCA7-HA-3a, pCA7-HA-3a-CS, or pCA7 control vector together with 0.5 μg of pCA7-NLRP3. At 24 h post-transfection, cells were fixed and permeabilized with 4% paraformaldehyde and 1% Triton X-100/PBS. After washing and blocking, the cells were incubated with rabbit anti-HA (561, MBL) and mouse anti-NLRP3 (Cryo-2; AdipoGen) antibodies, followed by Alexa Fluor 488-conjugated goat anti-rabbit IgG (H+L) and Alexa Fluor 568-conjugated goat anti-mouse IgG (H+L) (Life Technologies). Fluorescent signals were observed by confocal microscopy (A1R^+^, Nikon).

### Statistical Analysis

Statistical significance was tested using a two-tailed Student’s *t*-test. *P-*values < 0.05 were considered statistically significant.

## Results

### Viroporin 3a of SARS-CoV Is Sufficient to Stimulate IL-1β Secretion

**FIGURE 1:**
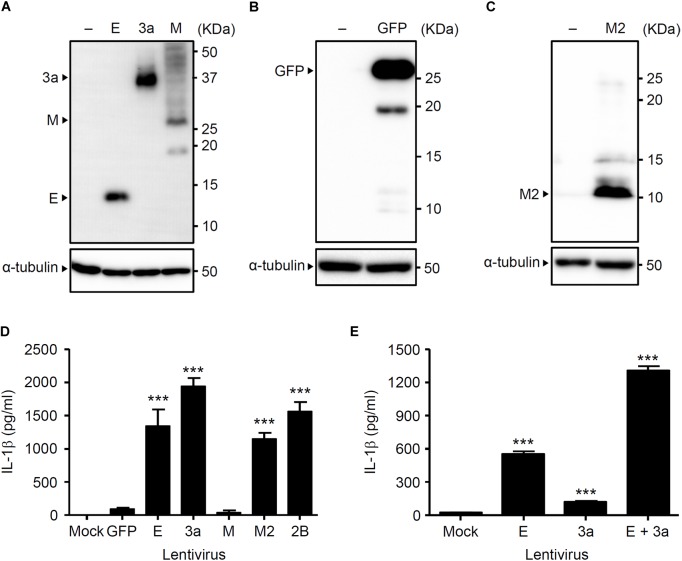
The 3a protein of SARS-CoV stimulates IL-1β secretion. **(A–C)** HEK293FT cells were transfected with pLenti6-E-V5, pLenti6-3a-V5, pLenti6-M-V5 **(A)**, pLenti-GFP-V5 **(B)**, or pLenti-M2-V5 plasmids **(C)**. Samples were analyzed by immunoblot with mouse monoclonal antibodies against V5-tag **(A)**, GFP **(B)**, or influenza virus M2 **(C)**. **(D,E)** LPS-primed BMM were infected with the lentivirus expressing SARS-CoV E, 3a, M, influenza virus M2, or EMCV 2B at MOI 0.25 **(D)** or 0.1 **(E)**. Supernatants were collected at 24 h post-infection and analyzed for IL-1β by ELISA. Data are representative of at least three independent experiments, and indicate the mean ± SD **(D,E)**; ^∗∗∗^*P* < 0.001.

We previously demonstrated that the influenza virus M2 protein (a proton-selective ion channel), its H37G mutant (which has lost its proton selectivity and enables the transport of other cations such as Na^+^ and K^+^), and the EMCV 2B protein (a Ca^2+^ channel) stimulates NLRP3 inflammasome-mediated IL-1β secretion (Ichinohe et al., 2010; Ito et al., 2012). In addition, the SARS-CoV E protein acts as a Ca^2+^-permeable ion channels that activates the NLRP3 inflammasome (Nieto-Torres et al., 2015). The fact that 3a protein of SARS-CoV acts as viroporin prompted us to examine whether it also triggers inflammasome activation. Thus, we first generated lentivirus plasmids expressing V5-tagged proteins and confirmed their expression in HEK293FT cells by immunoblot analysis (Figure 1A–C). We next transduced lipopolysaccharide (LPS)-primed BMMs with the lentiviruses expressing the SARS-CoV E, 3a, M, influenza virus M2, or EMCV 2B proteins. Consistent with previous reports (Ichinohe et al., 2010; Ito et al., 2012), IL-1β was released from LPS-primed BMMs transduced with the M2- and 2B-expressing lentivirus (Figure 1D). Similarly, the lentiviruses expressing the SARS-CoV E or 3a proteins stimulated IL-1β release from LPS-primed BMMs (Figure 1D). Furthermore, IL-1β secretion from LPS-primed BMMs co-infected with E- and 3a-expressing lentiviruses was significantly higher than that from SARS-CoV E-expressing lentivirus-infected cells (Figure 1E). These data indicated that the expression of SARS-CoV viroporin 3a is sufficient to stimulate IL-1β secretion by LPS-primed BMMs.

### The Ion Channel Activity of the 3a Protein Is Required for Inflammasome-Mediated IL-1β Secretion

**FIGURE 2:**
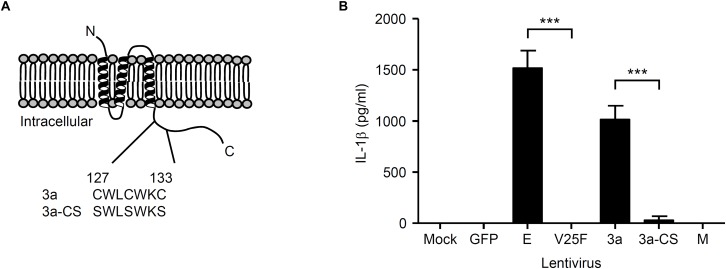
Ion channel activity of the 3a protein is required for IL-1β secretion. **(A)** SARS-CoV 3a protein; below, amino acid sequence of cysteine-rich domain (residue 127–133) of wild-type 3a and 3a-CS mutant. **(B)** LPS-primed BMM were infected with the lentivirus expressing SARS-CoV E, V25F, 3a, 3a-CS, or M at MOI 0.25. Supernatants were collected at 24 h post-infection and analyzed for IL-1β by ELISA. Data are representative of at least three independent experiments, and indicate the mean ± SD **(B)**; ^∗∗∗^*P* < 0.001.

Previous studies demonstrated that the N-terminal 40 amino acids of the SARS-CoV E protein are important for ion channel formation, and that mutations N15A and V25F [located in the transmembrane domain (from amino acid residues 7–38)] prevent ion conductivity (Wilson et al., 2004; Torres et al., 2007; Verdia-Baguena et al., 2012). In addition, the SARS-CoV 3a protein contains a cysteine-rich domain (amino acid residues 127–133) that is involved in the formation of a homodimer to generate the ion channel (Lu et al., 2006; Chan et al., 2009). Thus, mutation of the cysteine-rich domain blocks the ion conductivity by the 3a protein (Chan et al., 2009). To this end, we substituted amino acids Cys-127, Cys-130, and Cys-133 within the cysteine-rich domain of the SARS-CoV 3a protein with serine to generate a lentivirus expressing the ion channel activity-loss mutant, 3a-CS (Chan et al., 2009; Figure 2A). To test whether the ion channel activity of the SARS-CoV 3a protein is required to stimulate secretion of IL-1β, we transduced LPS-primed BMMs with lentiviruses expressing the SARS-CoV E, V25F, 3a, 3a-CS, or M proteins. Consistent with a previous report (Nieto-Torres et al., 2015), we found that the V25F mutant lentivirus failed to stimulate IL-1β release from BMMs (Figure 2B). Notably, the 3a-CS mutant completely abrogated IL-1β secretion (Figure 2B), suggesting that the ion channel activity of the 3a protein is required for SARS-CoV 3a-induced IL-1β secretion.

### SARS-CoV 3a Is Sufficient to Trigger Activation of the NLRP3 Inflammasome

**FIGURE 3:**
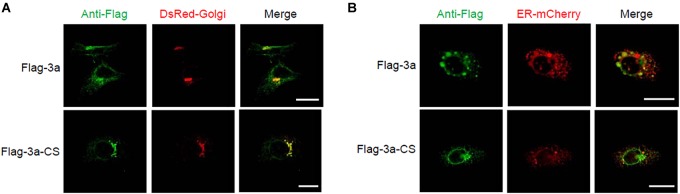
Subcellular localization of SARS-CoV 3a protein and 3a-CS mutant. **(A,B)** HeLa cells were transfected with the expression plasmid encoding flag-tagged 3a or 3a-CS and that encoding either DsRed-monomer-Golgi **(A)** or ER-mCherry **(B)**, and observed with a confocal microscope at 24 h post-transfection. Scale bars, 10 μm. Data are representative of at least three independent experiments.

Next, we determined the subcellular localization of the SARS-CoV 3a protein using confocal microscopy. When the SARS-CoV 3a protein was expressed in HeLa cells, we observed two main distribution patterns. Consistent with previous reports (Yu et al., 2004; Yuan et al., 2005), the 3a protein localized to the Golgi apparatus (Figure 3A). In addition, the 3a proteins concentrated in spot structures, which mainly localized to the endoplasmic reticulum (ER) (Figure 3B). By contrast, the 3a-CS mutant was concentrated in the Golgi apparatus rather than in the ER and did not form spot structures (Figure 3A,B).

**FIGURE 4:**
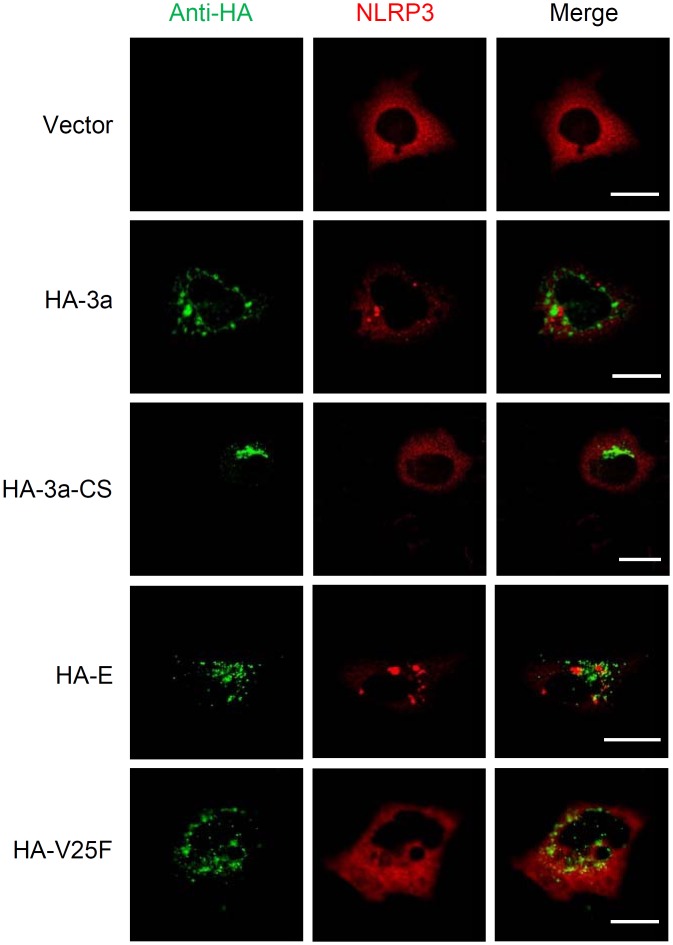
NLRP3 inflammasome activation by SARS-CoV 3a. HeLa cells were transfected with the expression plasmid encoding NLRP3 and that encoding HA-tagged SARS-CoV 3a, 3a-CS, E, or V25F, and by with a confocal microscope. Scale bars, 10 μm. Data are representative of at least three independent experiments.

We next examined the intracellular localization of NLRP3. Activation of the NLRP3 inflammasome led to a redistribution from the cytosol to the perinuclear space, a process considered as a hallmark of NLRP3 activation (Zhou et al., 2011; Ito et al., 2012; Johnson et al., 2013; Moriyama et al., 2016). Although cells expressing the ion channel activity-loss mutants 3a-CS or V25F uniformly expressed NLRP3 throughout the cytoplasm, it was redistributed to the perinuclear region in SARS-CoV 3a- or E-expressing cells (Figure 4). Together, these data provide evidence that the ion channel activity of the SARS-CoV 3a protein is essential for triggering the NLRP3 inflammasome.

### Both K^+^ Efflux and ROS Production Are Involved in the IL-1β Release Induced by the SARS-CoV 3a Protein

**FIGURE 5:**
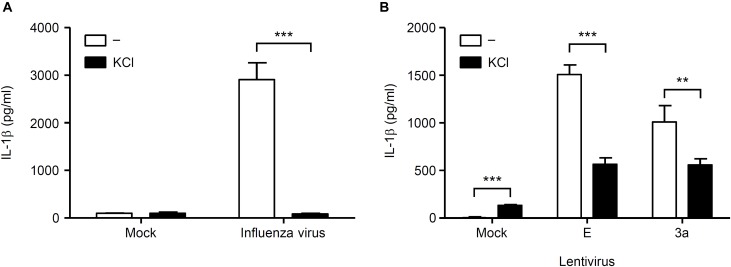
K^+^ efflux is required for activation of the NLRP3 inflammasome by SARS-CoV 3a protein. **(A,B)** BMMs were infected with influenza virus A/PR8 **(A)** or lentiviruses expressing SARS-CoV 3a or E proteins **(B)** and cultured in the presence or absence of KCl (130 mM). Cell-free supernatants were collected at 24 h post-infection, and analyzed for IL-1β by ELISA. Data are representative of at least three independent experiments, and indicate the mean ± SD; ^∗∗^*P* < 0.01 and ^∗∗∗^*P* < 0.001.

**FIGURE 6:**
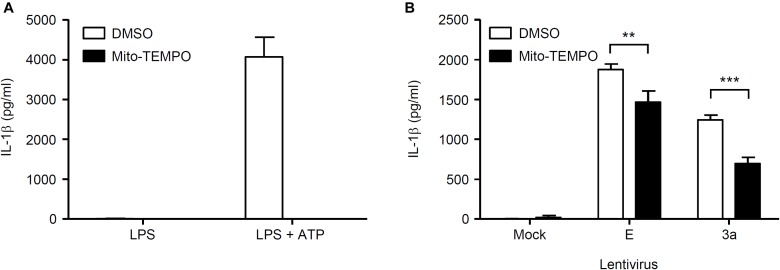
Mitochondrial ROS-dependent activation of the NLRP3 inflammasome by SARS-CoV 3a protein. **(A,B)** LPS-primed BMMs were stimulated with ATP **(A)** or lentiviruses expressing SARS-CoV 3a or E proteins **(B)** in the presence or absence of Mito-TEMPO (500 μM). Cell-free supernatants were collected at 24 h (lentiviruses) or 6 h (ATP) post-infection or stimulation, and analyzed for IL-1β by ELISA. Data are representative of at least three independent experiments, and indicate the mean ± SD; ^∗∗^*P* < 0.01 and ^∗∗∗^*P* < 0.001.

Finally, we investigated the mechanism by which SARS-CoV 3a triggers NLRP3 inflammasome activation. A previous study showed that the 3a protein of SARS-CoV acts as a K^+^ channel (Lu et al., 2006). In addition, K^+^ efflux is a well-known activator of the NLRP3 inflammasome (Mariathasan et al., 2006; Petrilli et al., 2007). These observations prompted us to examine whether K^+^ efflux is required for 3a-mediated IL-1β secretion. To this end, BMMs in K^+^-rich medium were infected with influenza A virus or lentiviruses expressing the SARS-CoV E or 3a proteins. In agreement with a previous result (Ichinohe et al., 2010), we found that IL-1β secretion caused by influenza virus was completely blocked when the extracellular K^+^ concentration was increased to 130 mM (Figure 5A). The inhibitory effect of the K^+^-rich medium was also observed when cells were stimulated with lentiviruses expressing the SARS-CoV E or 3a proteins (Figure 5B). Since mitochondrial ROS are important for NLRP3 inflammasome activation (Nakahira et al., 2011; Zhou et al., 2011), we next stimulated BMMs with extracellular ATP or lentiviruses expressing the SARS-CoV E or 3a proteins in the presence or absence of the antioxidant, Mito-TEMPO, a scavenger that is specific for mitochondrial ROS (Jiang et al., 2009; Trnka et al., 2009). As reported previously (Nakahira et al., 2011; Ito et al., 2012), treatment of BMMs with Mito-TEMPO completely blocked IL-1β secretion in response to ATP (Figure 6A). Similarly, IL-1β release induced by the SARS-CoV E and 3a proteins was significantly inhibited by Mito-TEMPO (Figure 6B). These observations indicate that the SARS-CoV 3a protein disrupts intracellular ionic concentrations and causes mitochondrial damages, thereby activating the NLRP3 inflammasome.

## Discussion

In summary, we found that the ion channel activity of SARS-CoV 3a protein is essential for activation of the NLRP3 inflammasome. In addition, both K^+^ efflux and mitochondrial ROS production are required for SARS-CoV 3a-mediated IL-1β secretion.

Thus far, several models have been proposed to explain NLRP3 inflammasome activation by RNA viruses. First, viral RNA or RNA cleavage products generated by RNase L activate the NLRP3 inflammasome via the DExD/H-box helicase, DHX33 (Allen et al., 2009; Mitoma et al., 2013; Chen et al., 2014; Chakrabarti et al., 2015). Second, viroporins encoded by RNA viruses activates the NLRP3 inflammasome (Ichinohe et al., 2010; Ito et al., 2012; Triantafilou et al., 2013; Nieto-Torres et al., 2015). In the case of influenza virus, the proton-selective M2 ion channel in the acidic *trans*-Golgi network activates the NLRP3 inflammasome (Ichinohe et al., 2010). Interestingly, an M2 mutant in which histidine was substituted with glycine at position 37 (H37G), causing loss of proton selectivity, enables transport of other cations (i.e., Na^+^ and K^+^), thereby leading to enhanced secretion of IL-1β from LPS-primed BMMs and dendritic cells when compared with the wild-type M2 protein. In addition, the 2B proteins of EMCV, poliovirus, enterovirus 71 (EV71), and human rhinovirus (a member of the *Picornaviridae* family) triggers NLRP3 inflammasome activation by inducing Ca^2+^ flux from the ER and Golgi compartments (Ito et al., 2012; Triantafilou et al., 2013). Furthermore, hepatitis C virus stimulates NLRP3 inflammasome-mediated IL-1β production though its p7 viroporin (Negash et al., 2013; Farag et al., 2017). Third, a recent study has demonstrated that the 3D protein of EV71 directly interacts with NLRP3 to facilitate the assembly of NLRP3 inflammasome complex (Wang et al., 2017).

In the case of SARS-CoV, the viroporin E forms forms Ca^2+^-permeable ion channels and activates the NLRP3 inflammasome (Nieto-Torres et al., 2015). In addition, another viroporin 3a was found to induce NLRP3 inflammasome activation (Yue et al., 2018). Although alanine substitution at Cys-133, which is required for dimer or tetramer formation (Lu et al., 2006), still allows activation of the NLRP3 inflammasome by interacting with caspase-1 (Yue et al., 2018), the ion channel activity-loss mutant 3a-CS (Cys-to-Ser substitution at positions Cys-127, Cys-130, and Cys-133) (Chan et al., 2009) completely abrogated IL-1β secretion from LPS-primed BMMs, suggesting that the 3a protein of SARS-CoV has the ability to induce the NLRP3 inflammasome activation by multiple mechanisms. Previous studies show that the 3a protein of SARS-CoV is localized to the plasma membrane (Minakshi and Padhan, 2014) and acts as a K^+^ channel (Lu et al., 2006), thereby (presumably) stimulating the K^+^ efflux at the plasma membrane. Indeed, we found that IL-1β secretion caused by the 3a protein was significantly inhibited when the extracellular K^+^ concentration increased to 130 mM. Although it remains unclear whether another viroporin 8a of SARS-CoV (Castano-Rodriguez et al., 2018) activates the NLRP3 inflammasome, these data highlights the importance of viroporins in SARS-CoV-induced NLRP3 inflammasome activation. A better understanding of the mechanism that governs the NLRP3 inflammasome will facilitate the development of more effective interventions for the treatment of infectious diseases and increase our understanding of viral pathogenesis.

## Author Contributions

I-YC and TI designed the study and wrote the manuscript. I-YC and MM performed the experiments. I-YC, MM, and TI analyzed the data. M-FC provided reagents and advice. All authors reviewed the manuscript.

## Conflict of Interest Statement

The authors declare that the research was conducted in the absence of any commercial or financial relationships that could be construed as a potential conflict of interest.
